# Regulation of factor V and factor V-short by TFPIα: Relationship between B-domain proteolysis and binding

**DOI:** 10.1074/jbc.RA120.016341

**Published:** 2021-01-07

**Authors:** Teodolinda Petrillo, Francis Ayombil, Cornelis van’t Veer, Rodney M. Camire

**Affiliations:** 1 Division of Hematology and the Raymond G. Perelman Center for Cellular and Molecular Therapeutics, The Children’s Hospital of Philadelphia, Philadelphia, Pennsylvania, USA; 2 Center of Experimental and Molecular Medicine, Amsterdam UMC, University of Amsterdam, Amsterdam, the Netherlands; 3 Department of Pediatrics, Perelman School of Medicine, University of Pennsylvania, Philadelphia, Pennsylvania, USA

**Keywords:** factor V, tissue factor pathway inhibitor, factor Va, prothrombinase, coagulation factor, thrombin, protein complex, hemostasis, prothrombin, AR2, acidic region 2, BR, basic region, BSA, bovine serum albumin, DAPA, Dansylarginine-N-(3-ethyl-1,5-pentanediyl)amide, EDTA, ethylenediaminetetraacetic acid, FV, factor V, FV-BR, fragment containing the BR sequence of FV, FV-DP, plasma deficient in FV, FVa, activated FV, FXa, activated factor X, HC, heavy chain, K1, K2, K3, Kunitz domain 1, 2, 3 of TFPIα, LC, light chain, OG_488_-TFPIα-BR, TFPIα-BR modified with Oregon Green_488_ maleimide, PBS, phosphate-buffered saline, PCPS, small unilamellar vesicles containing 75% (w/w) L-α-phosphatidylcholine and 25% (w/w) L-α-phosphatidylserine, PD-FV, plasma-derived FV, RVV-V, FV-activating proteinase from Russell’s viper venom, TFPIα, tissue factor pathway inhibitor alpha, TFPIα-BR, fragment containing the BR sequence of TFPIα

## Abstract

Coagulation factor V (FV) plays an anticoagulant role but serves as a procoagulant cofactor in the prothrombinase complex once activated to FVa. At the heart of these opposing effects is the proteolytic removal of its central B-domain, including conserved functional landmarks (basic region, BR; 963–1008 and acidic region 2, AR2; 1493–1537) that enforce the inactive FV procofactor state. Tissue factor pathway inhibitor α (TFPIα) has been associated with FV as well as FV-short, a physiologically relevant isoform with a shortened B-domain missing the BR. However, it is unclear which forms of FV are physiologic ligands for TFPIα. Here, we characterize the binding and regulation of FV and FV-short by TFPIα *via* its positively charged C-terminus (TFPIα-BR) and examine how bond cleavage in the B-domain influences these interactions. We show that FV-short is constitutively active and functions in prothrombinase like FVa. Unlike FVa, FV-short binds with high affinity (K_d_ ∼1 nM) to TFPIα-BR, which blocks procoagulant function unless FV-short is cleaved at Arg^1545^, removing AR2. Importantly, we do not observe FV binding (μM detection limit) to TFPIα. However, cleavage at Arg^709^ and Arg^1018^ displaces the FV BR, exposing AR2 and allowing TFPIα to bind *via* its BR. We conclude that for full-length FV, the detachment of FV BR from AR2 is necessary and sufficient for TFPIα binding and regulation. Our findings pinpoint key forms of FV, including FV-short, that act as physiologic ligands for TFPIα and establish a mechanistic framework for assessing the functional connection between these proteins.

The proteolytic conversion of factor V (FV) to activated FV (FVa) is central to the amplification of coagulation and necessary for blood clot formation (1). FV circulates in the blood as a single-chain, inactive procofactor in which the N-terminal heavy chain (HC; domains A1-A2) and C-terminal light chain (LC; domains A3-C1-C2) are situated between a central B-domain (Fig. 1*A*) (1, 2). Proteolysis of FV at Arg^709^, Arg^1018^, and Arg^1545^ by thrombin or factor Xa (FXa) releases the B-domain and activates it to FVa (3, 4, 5, 6, 7, 8). As a cofactor, FVa binds membrane-bound FXa with high affinity forming the prothrombinase complex, which converts prothrombin to thrombin (9). FVa is essential for thrombin generation as it increases the rate at which FXa acts on prothrombin by several orders of magnitude (10).

**Figure 1:**
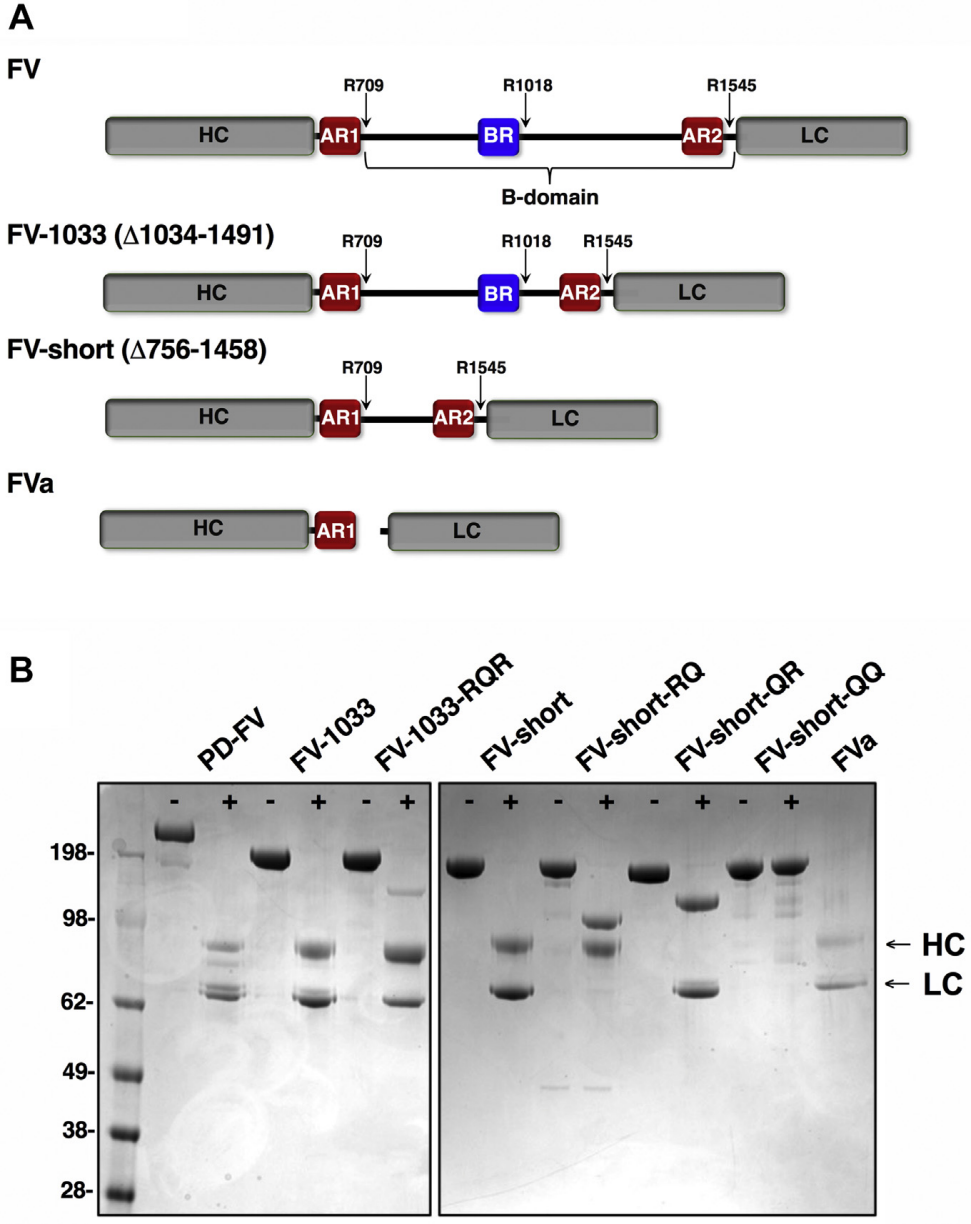
**FV species and quality control.***A*, schematic representation of FV species used in this study. The B-domain, defined by residues 710 to 1545 lies between the HC and LC. FV-1033 has B-domain residues 1034 to 1491 deleted and FV-short has residues 756 to 1458 deleted. Thrombin cleavage sites are indicated as are AR1, AR2, and the BR. *B*, purified FV species (300 nM) before (−) and after (+) treatment with thrombin (2 nM) or RVV-V (2 nM for FV-short-QR), were subject to SDS-PAGE (4–12% gradient) under reducing conditions and stained with Coomassie Blue R-250. Bands corresponding to the HC and LC are indicated on the right. FV mutants in which specific thrombin cleavage sites were changed from Arg to Gln are indicated.

Insights into the mechanism by which FV is kept as a procofactor and how proteolysis in the B-domain affects FV activation have been made (2, 11, 12, 13, 14). Evolutionary conserved regions in the B-domain called the basic region (BR; residues 963–1008) and acidic region 2 (AR2; residues 1493–1537) work together by blocking the FXa binding site on the HC/LC (Fig. 1*A*) (15, 16). The BR+AR2 make up the minimal sequences necessary to keep FV inactive. For example, elimination of most of the B-domain, except the BR+AR2, yields a FV derivative that is maintained as a procofactor (16). However, removal of either the BR or AR2 by recombinant deletion produces a constitutively active derivative, which functions like FVa (15, 16). Consistent with this model, an exogenous FV-BR fragment binds with high affinity to B-domainless forms of FV that retain AR2, but lack the BR (*e.g.*, FV-810; Δ811–1491). The binding of this FV-BR fragment to FV-810 restricts FXa binding and blocks procoagulant activity (17).

The importance of these functional landmarks in the B-domain goes beyond maintaining FV as a procofactor. For example, tissue factor pathway inhibitor alpha (TFPIα) binds to an exposed AR2 on partially proteolyzed forms of FV and inhibits procoagulant function by preventing assembly in prothrombinase (18, 19). Remarkably, the positively charged C-terminal tail of TFPIα (TFPIα-BR) is homologous to FV-BR and mediates binding to AR2 (18, 20). Platelet-derived FV, which is proteolyzed in the B-domain and secreted from α-granules, and plasma FV, partially cleaved by FXa or thrombin, represent potential physiologic forms that may be regulated by TFPIα (18, 21). These forms of FV lack the BR yet retain AR2 (herein referred to as FV_AR2_) and are likely present during the initiation phase of coagulation. Thus, in addition to inhibiting FXa and FVIIa-tissue factor (TF) (22), TFPIα may also contribute to regulating thrombin generation at the level of prothrombinase (18, 23).

Additional connections between FV and TFPIα have been made with potentially important implications. Duckers *et al.* (24) found that the variable and milder than expected bleeding phenotype associated with FV deficiency is likely related to low levels of TFPIα found in FV-deficient plasma. In that study, plasma concentrations of FV and TFPIα were found to correlate and immunoadsorption of FV from plasma-depleted TFPIα suggesting they form a complex. Consistent with this, FV and TFPIα interact using ligand blot methods, and surface plasmon resonance (SPR) studies found that plasma-derived FV (PD-FV) bound immobilized TFPIα with an estimated affinity of 13 nM (24, 25). Additionally, it has been reported that PD-FV enhances TFPIα-mediated inhibition of membrane-bound FXa (19, 26); however, this effect was not seen with recombinant FV (rFV) (27). Lastly, TFPIα *via* its BR impedes FV activation by thrombin or FXa by delaying cleavage at Arg^1545^ (28). There is also some evidence that FVa interacts with TFPIα as it enhances TFPIα inhibition of membrane-bound FXa (19, 29), and the proteins appear to bind using different methods (25, 30).

A fascinating advance in the TFPIα-FV axis came from the identification of a splicing isoform of FV (31). Recognized in a family with a moderately severe bleeding disorder (East Texas bleeding disorder), the mutation in the *F5* gene (A2440G; S756G) activates a weak splice site in exon 13 resulting in an abundant, alternatively spliced transcript (32, 33). It encodes for a new FV isoform called FV-short, which lacks 702 amino acids (Δ756–1458) in the B-domain including the BR but retains AR2 (Fig. 1*A*). Family members with the mutation have high plasma levels of FV-short (∼2–5 nM) and remarkably have elevated TFPIα (tenfold) (33). It was shown that FV-short and TFPIα form a complex in plasma, likely *via* the exposed AR2 on FV-short (33). The increase in plasma TFPIα is thought to contribute to the bleeding phenotype and likely results from a change in the way TFPIα is cleared from the circulation when bound to FV-short (34). At least two other genetic changes in exon 13 from different families have been identified that enhance splicing and produce a FV-short protein with parallel increases in TFPIα (35, 36). Importantly, in normal controls splicing occurs within exon 13 at a low level, as FV-short is present in normal plasma at subnanomolar concentration (33). The importance of FV-short on normal hemostasis is not known, but a recent report found it enhances the TFPIα-cofactor activity of protein S with respect to FXa inhibition (37).

Despite these advances, there are inconsistencies making it unclear which forms of FV are true physiologic ligands for TFPIα. This is an important question to answer as it has a bearing on understanding key regulatory points in coagulation impacted by FV/TFPIα. This information could guide potential strategies targeting this molecular interaction for therapeutic benefit. Additionally, despite its potential significance in the coagulation system, FV-short and its binding to TFPIα have yet to undergo biochemical characterization. Here, we assess the binding and regulation of FV and FV-short by TFPIα and examine how cleavage in the B-domain influences these interactions. Our findings show that FV and FVa are not physiologic ligands for TFPIα. Rather, TFPIα binds and regulates forms of FV that lack the internal BR such as FV cleaved at Arg^709^/Arg^1018^ and FV-short. The studies provide new mechanistic insight into the regulation of coagulation and uncover the necessary transformation that FV must undergo to interact with TFPIα.

## Results

### Protein preparation

A schematic representation of FV species used is shown in Figure 1*A*. FV was purified from the plasma while other FV species were expressed and purified in milligram quantities. Due to its robust expression compared with rFV, a FV derivative with a shortened B-domain, FV-1033 (Δ1034–1491), and a variant FV-1033-RQR (Arg^1018^ changed to Gln) were prepared. FV-short and mutants with Gln at one or both thrombin cleavage sites (Arg^709^ and/or Arg^1545^; FV-short-RQ, FV-short-QR, and FV-short-QQ) were also purified. The mutations render those cleavage sites uncleavable. Proteins migrated at the expected position on reduced SDS-PAGE (Fig. 1*B*). Thrombin cleavage of PD-FV, FV-1033, FV-1033-RQR, and FV-short yielded the expected HC and LC similar to FVa. FV-short-RQ and FV-short-QR were proteolytically processed only at Arg^709^ and Arg^1545^, respectively, while FV-short-QQ was not cleaved by thrombin (Fig. 1*B*).

### FV-short is constitutively active

**Figure 2:**
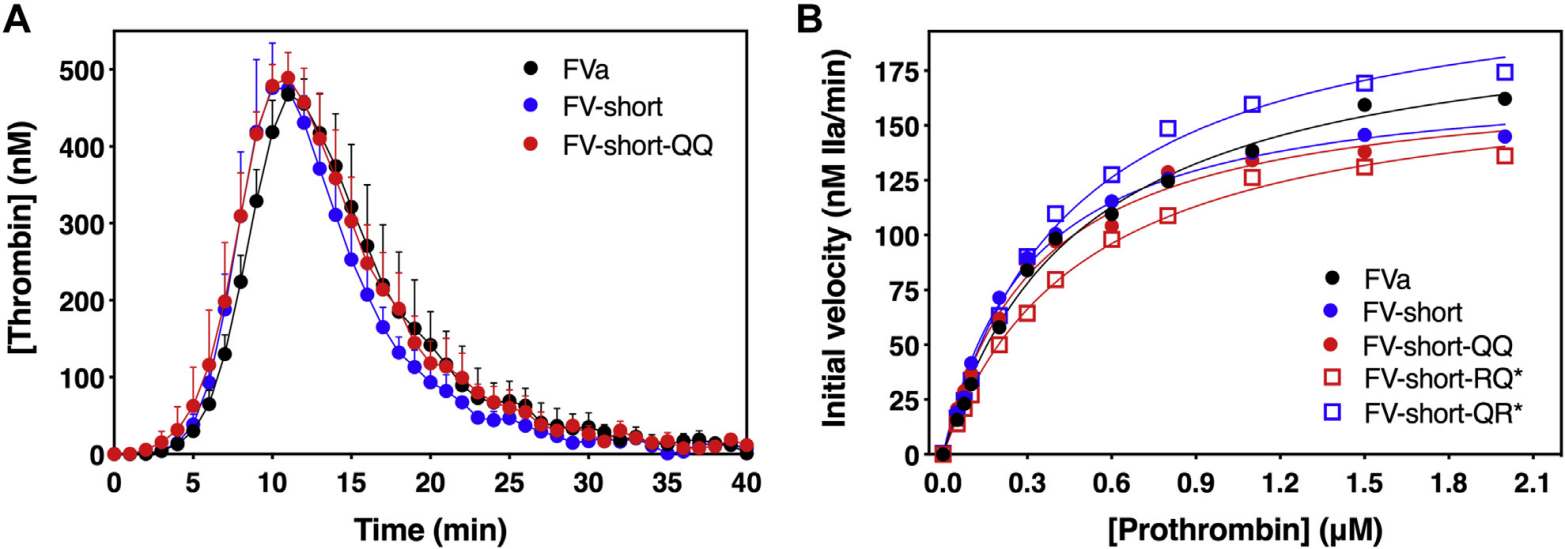
**FV-short is constitutively active like FVa.***A*, thrombin generation was measured in FV-DP supplemented with 1.5 nM FV-short, FV-short-QQ, or FVa and initiated with 0.4 pM TF. The data show the average of three independent experiments with standard deviation. *B*, prothrombin activation by prothrombinase assembled with FVa or different FV-short mutant. The (∗) refers to FV-short mutants precleaved prior to introduction into the assay. The initial velocity of thrombin generation was determined at increasing concentrations of prothrombin and data were fit using the Michaelis–Menten equation using GraphPad Prism (v.9.0). For the data in *B*, the fitted constants and the standard errors are: FVa, K_M_ = 0.48 ± 0.04 μM, V_max_ = 204.0 ± 6.0 nM thrombin/min; FV-short, K_M_ = 0.31 ± 0.02 μM, V_max_ = 173.4 ± 4.2 nM thrombin/min; FV-short-QQ, K_M_ = 0.34 ± 0.03 μM, V_max_ = 172.4 ± 4.5 nM thrombin/min; FV-short-RQ∗, K_M_ = 0.49 ± 0.03 μM, V_max_ = 175.3 ± 4.0 nM thrombin/min; and FV-short-QR∗, K_M_ = 0.46 ± 0.04 μM, V_max_ = 222.7 ± 7.3 nM thrombin/min. The data are representative of 3 to 6 independent experiments. The average value for the kinetic constants and standard deviations are summarized in Table 2.

Using a prothrombin time (PT)-based clotting assay, we found that FV-short and FV-short mutants exhibit specific activities similar to FVa (Table 1). Precleavage of FV-short-RQ at Arg^709^ with thrombin (FV-short-RQ∗) or FV-short-QR at Arg^1545^ with RVV-V (FV-short-QR∗) prior to addition into the clotting assay did not alter their activity. Further, FV-short, FV-short-QQ, and FVa exhibited similar thrombin generation profiles in a thrombin generation assay (TGA; Fig. 2*A*). As expected, the procofactors, PD-FV and FV-1033, have low specific activities in the clotting assay, which was similar to the FV activity contained in pooled normal plasma (PNP)†† (Table 1). Using a purified component assay, the kinetic constants for prothrombin activation using FV-short, FV-short-QQ, and cleaved FV-short mutants were similar to FVa (Fig. 2*B* and Table 2). Together, these data show that FV-short is constitutively active like FVa and does not require cleavage at Arg^709^ or Arg^1545^ to express any further activity making it distinct from FV.

**Table 1:** Specific activity of FV species

FV species	Specific activity (*Units/nmol*)		
	Buffer	+ TFPIα-BR	+ FV-BR
FVa	325.3 ± 59.2	336.8 ± 32.5	342.3 ± 38.1
FV-short	282.7 ± 23.8	53.6 ± 7.1	72.1 ± 10.3
FV-short-QQ	324.2 ± 28.9	58.3 ± 10.8	76.0 ± 9.4
FV-short-RQ	289.9 ± 29.8	56.3 ± 8.1	99.2 ± 16.1
FV-short-QR	289.3 ± 22.1	71.4 ± 5.9	87.7 ± 11.0
FV-short-RQ∗	285.2 ± 15.0	83.0 ± 19.9	135.9 ±14.9
FV-short-QR∗	316.6 ± 41.6	291.2 ± 28.8	287.1 ± 52.3
PNP	55.9 ± 4.3	5.0 ± 0.7	9.2 ± 1.3
PD-FV	79.0 ± 6.4	6.7 ± 1.0	11.8 ± 3.8
FV-1033	38.1 ± 4.1	15.1 ± 3.2	21.2 ± 3.9
FV-1033-RQR	8.8 ± 0.9	1.9 ± 0.4	2.7 ± 0.2

The specific activity of FV species was determined using a PT-based clotting assay in FV-DP as described in “Experimental procedures.” The concentration of FV in PNP was assumed to be 20 nM, and the theoretical specific activity was calculated to be 50 U/nmol. The impact of 5 μM TFPIα-BR or FV-BR fragments on FV specific activity was also determined. The (∗) refers to FV-short mutants precleaved prior to introduction into the assay. Data represent the average of at least five measurements and the error represents the standard deviation.

**Table 2:** FV-short is constitutively active and is regulated by TFPIα

FV species	Prothrombin		TFPIα-BR	TFPIα
	K_M_ (μM)	V_max_ (nM thrombin/min)	K_i_ (nM)	
FVa	0.46 ± 0.05	183.8 ± 22.2	ND (>7 μM)	ND (>0.5 μM)
FV-short	0.40 ± 0.08	165.7 ± 11.6	23.6 ± 6.5	0.23 ± 0.08
FV-short-QQ	0.41 ± 0.08	167.4 ± 12.7	23.4 ± 1.7	0.11 ± 0.02
FV-short-RQ∗	0.53 ± 0.13	162.2 ± 36.4	83.9 ± 10.9	0.24 ± 0.04
FV-short-QR∗	0.69 ± 0.17	248.7 ± 14.6	ND (>7 μM)	ND (>0.5 μM)

Steady-state kinetic constants were determined using membrane-bound-FXa and the indicated cofactor species and analyzed using the Michaelis–Menten equation. K_i_ for the inhibition of prothrombinase by TFPIα-BR or TFPIα was determined using limiting concentrations of cofactor species and analyzed using the equation for competitive inhibition. For experiments with TFPIα, an anti-TFPIα-K2 domain antibody was added to block FXa inhibition and isolate the effects of TFPIα to its C-terminal region. The (∗) refers to FV-short mutants precleaved prior to introduction into the assay. Data are the average of at least 3–6 individual experiments and the error represents the standard deviation.

### TFPIα regulates FV-short function

FV-short is structurally distinct from FVa as a small region of the B-domain remains between the HC and LC, which includes AR2 (Fig. 1*A*). We first used clotting assays and TGA to examine the impact of TFPIα-BR on FV-short function in plasma. Since full-length TFPIα has multiple modes of inhibition in the plasma, we first used a recombinant TFPIα-BR fragment to simplify data interpretation.

TFPIα-BR had no effect on the specific activity of FVa in a clotting assay (Table 1). In contrast, the specific activities of FV-short and uncleaved mutants were decreased in the presence of TFPIα-BR. Prior cleavage of FV-short at Arg^709^ (FV-short-RQ∗) had only a weak effect on the ability of TFPIα-BR to inhibit FV-short cofactor function. However, TFPIα-BR had no effect on cofactor function if FV-short was precleaved at Arg^1545^ (FV-short-QR∗). Similar results were obtained using the homologous FV-BR fragment (Table 1). These data show that TFPIα-BR regulates FV-short function but only if AR2 is attached to the LC.

**Figure 3:**
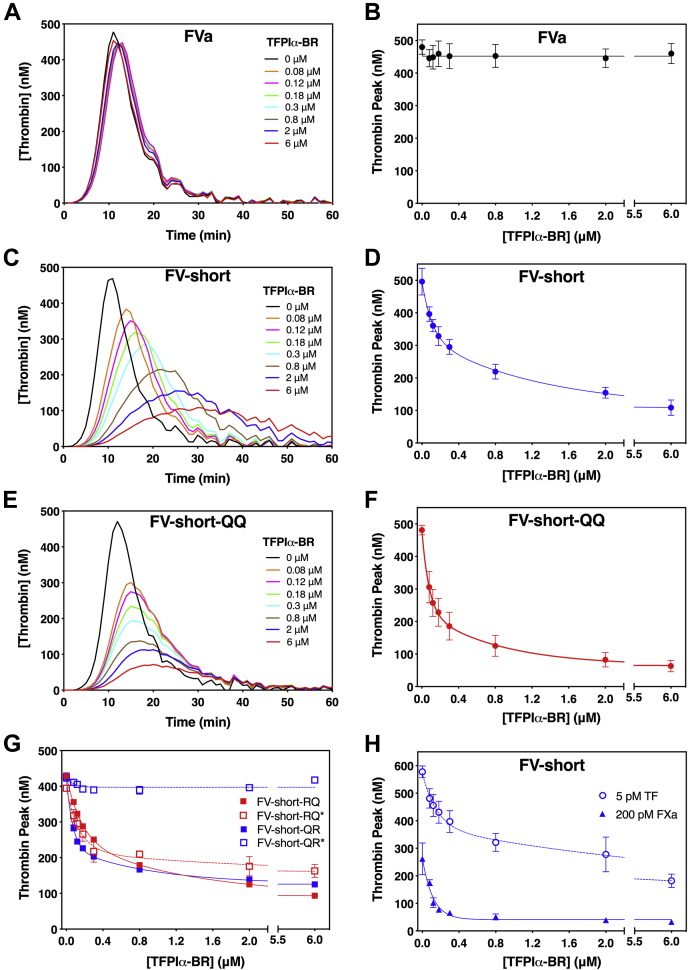
**TFPIα-BR regulates thrombin generation initiated with FV-short.** Thrombin generation was measured in FV-DP supplemented with 1.5 nM FVa (*A*–*B*), FV-short (*C* and *D*), or FV-short-QQ (*E* and *F*). *A*, *C* and *E* are thrombin generation curves with increasing amounts of TFPIα-BR initiated with 0.4 pM TF. *B*, *D*, *F* and *G* represent the change in thrombin peak height at different TFPIα-BR concentrations initiated 0.4 pM TF, while *H* employs different initiators. Cofactor species used in the assay are indicated on the graph. The (∗) refers to FV-short mutants precleaved prior to introduction into the assay. For *A*, *C*, and *E*, data are a single representative experiment that was performed three times. For *B*, *D*, *F*, *G*, and *H*, the data show the average thrombin peak with standard deviation from three independent experiments. In these graphs, the data were fit using a two-phase exponential decay model.

The TGA was used to gain additional insight into how TFPIα-BR impacts FV-short function. Consistent with the clotting assay, FVa was unaffected by TFPIα-BR (Fig. 3, *A* and *B*). FV-short was quite sensitive to TFPIα-BR inhibition (Fig. 3, *C* and *D*) using a TF trigger of 0.4 pM; however, TFPIα-BR had a more prominent inhibitory effect on FV-short-QQ (Fig. 3, *E* and *F*). We speculate that FV-short is less sensitive to TFPIα-BR compared with the QQ mutant because once coagulation is initiated, thrombin or FXa produced in the assay cleaves FV-short removing its B-domain, thereby relieving inhibition by TFPIα-BR. This relief of inhibition is related to cleavage at Arg^1545^, as FV-short precleaved at Arg^1545^ (FV-short-QR∗) was insensitive to TFPIα-BR while precleavage at Arg^709^ (FV-short-RQ∗) maintained inhibition (Fig. 3*G*). Further, if strong initiator conditions were used (TF, 5 pM), FV-short was less sensitive to TFPIα-BR compared with the results using a weaker initiator (FXa, 200 pM) (Fig. 3*H*). We speculate that TGA conditions, which promote robust thrombin generation, result in more rapid feedback proteolysis of FV-short at Arg^709^ and Arg^1545^, which eliminates binding to the C-terminal end of TFPIα.

**Figure 4:**
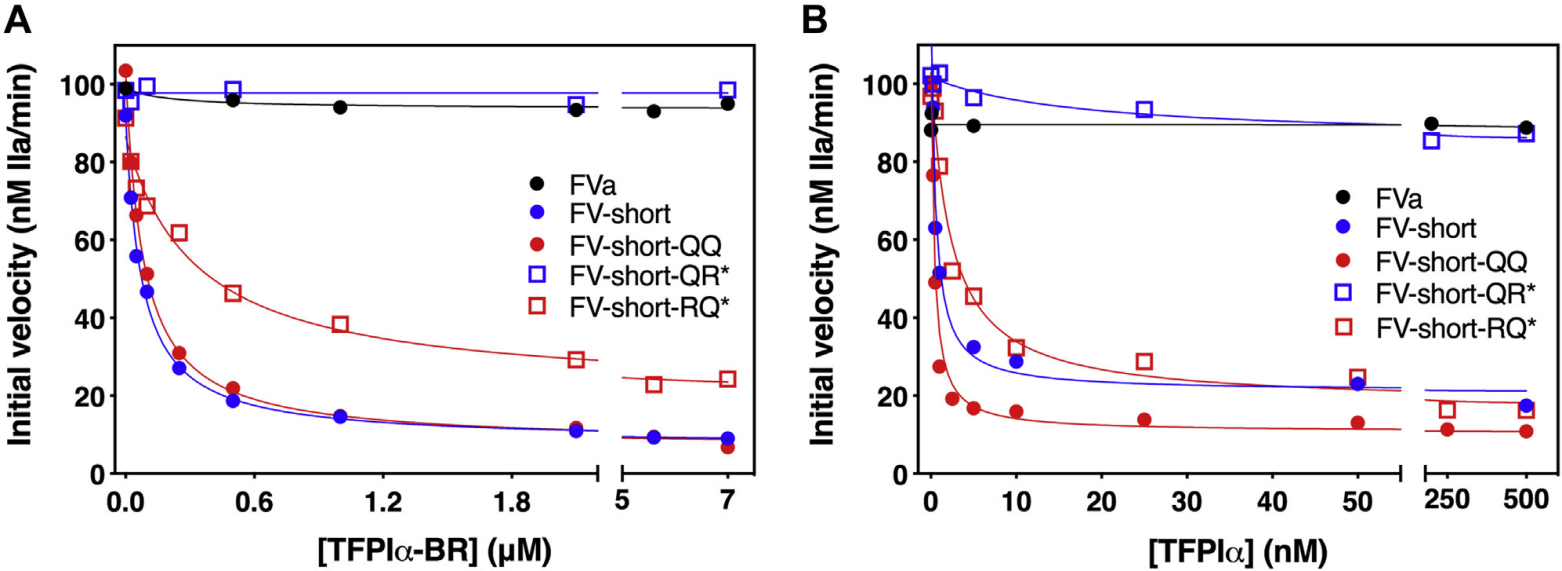
**TFPIα-BR and TFPIα inhibits prothrombin activation using FV-short but not FVa.** The initial velocity of thrombin generation was determined at increasing concentrations of TFPIα-BR (*A*) or TFPIα (*B*). Prothrombinase was assembled using limiting amounts of the cofactor species. In *B*, an excess of an anti-K2 antibody to block FXa inhibition was included in the assay. The (∗) refers to FV-short mutants precleaved prior to introduction into the assay. The data were fit to the equation for competitive inhibition using GraphPad Prism (v. 9.0). The calculated inhibition constants and the standard errors are: for *A*) FV-short, K_i_ = 19.8 ± 0.1 nM; FV-short-QQ, K_i_ = 21.5 ± 0.1 nM; FV-short-RQ∗, K_i_ = 86.1 ± 14.1 nM; for data in *B*) FV-short, K_i_ = 0.14 ± 0.03 nM; FV-short-QQ, K_i_ = 0.10 ± 0.02 nM; FV-short-RQ∗, K_i_ = 0.26 ± 0.03 nM. No inhibition constant could be calculated for FVa or FV-short-QR∗. The data are representative of 3 to 5 independent experiments. The average value for the inhibition constants and standard deviations are summarized in Table 2.

We next assessed the sensitivity of FV-short cofactors to full-length TFPIα or TFPIα-BR in a prothrombin activation assay. In this assay, the FXa inhibitory activity of TFPIα was blocked with an anti-Kunitz domain 2 (K2) antibody. FV-short or FV-short-QQ was strongly inhibited by TFPIα-BR with a K_i_ of ∼25 nM while FVa and FV-short-QR∗ were not (Fig. 4*A*, Table 2). Interestingly, FV-short-RQ∗ had a threefold reduced sensitivity toward TFPIα-BR (Table 2). Using full-length TFPIα, similar trends in the data were seen except the inhibition constants were lower (Fig. 4*B* and Table 2). Like TFPIα-BR, full-length TFPIα did not inhibit prothrombinase when using FVa as the cofactor. We speculate the difference in inhibitory potential for TFPIα-BR and full-length TFPIα could relate to some TFPIα not being fully inhibited by the anti-K2 antibody and/or to steric/structural differences between the two proteins, which impact inhibition of thrombin generation.

### TFPIα binds FV-short, not full-length FV

Prior work found that TFPIα and FV interact and form a complex in plasma (24, 25, 33). Indeed, PNP was sensitive to added TFPIα-BR as was purified PD-FV or FV-1033 in a clotting assay and TGA (Table 1, Fig. S1). While these data could suggest that TFPIα-BR binds and blocks the function of FV, this would be difficult to reconcile with our understanding of how FV’s internal BR engages with AR2. Either, TFPIα disrupts the intramolecular FV BR-AR2 interaction, or it binds another region of FV. An alternative possibility is that TFPIα does not bind FV, but rather engages and alters the function of intermediates formed during the assay including forms of FV with an exposed AR2 (*e.g.*, FV_AR2_).

**Figure 5:**
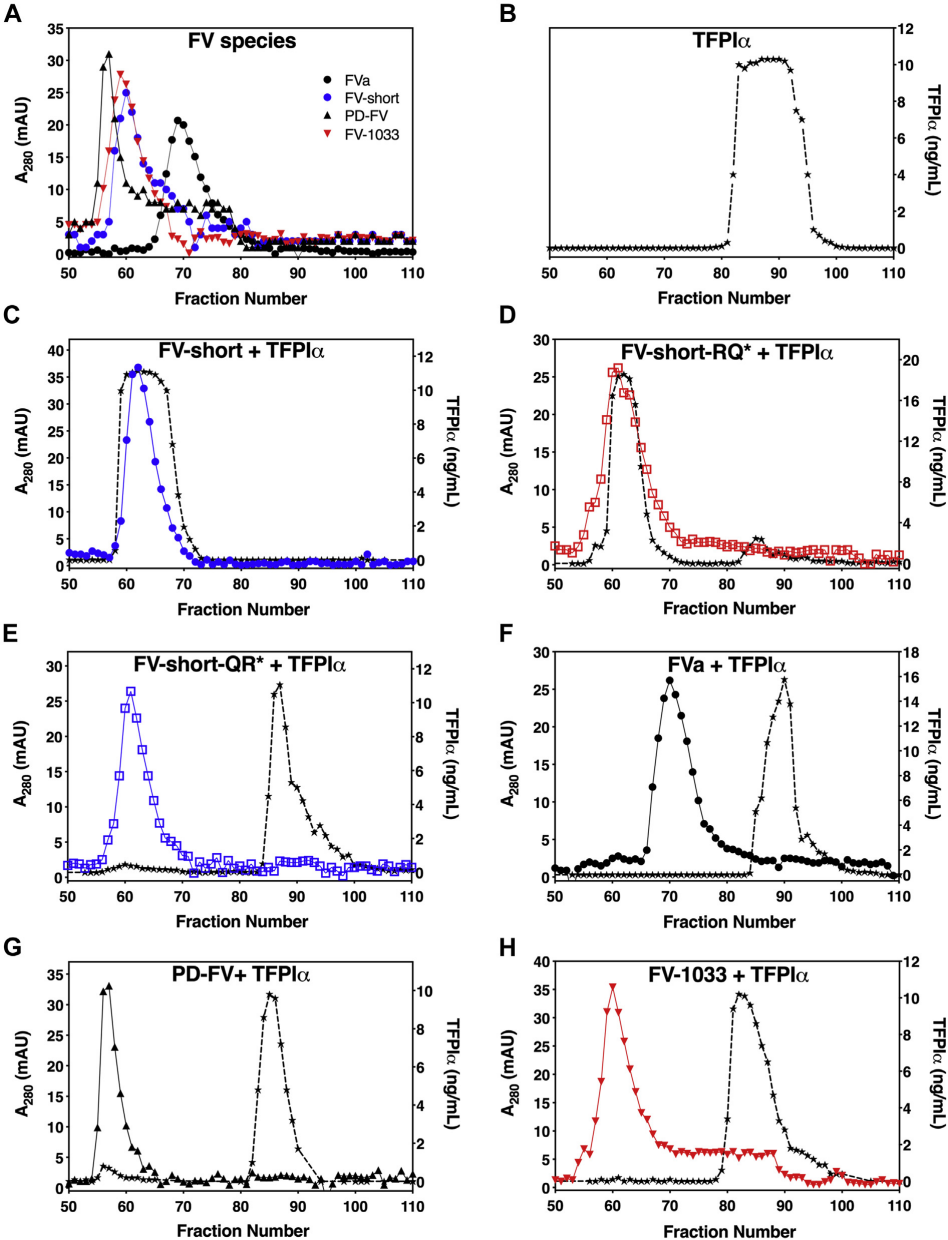
**TFPIα binds FV-short but not FV.** FV species (*A*) and TPFIα (*B*) alone or premixed at 1 μM each (*C*–*H*) were loaded onto a Sephacryl S-200 column in HBS/Ca^2+^, pH 7.4. Collected fractions were assessed for TFPIα by ELISA (right y-axis; *black star symbol* and *dashed black line*) or total protein as measured by absorbance at 280 nm (left y-axis). FV species used in the assay are indicated on the graph. The (∗) refers to FV-short mutants precleaved prior to introduction into the assay. The data are representative of at least two independent experimental runs.

To examine this, we first used size-exclusion chromatography (SEC) to qualitatively assess binding. Due to large differences in molecular weight, the elution profiles of individually loaded PD-FV (peak fraction 57), FV-1033 (peak fraction 60), FV-short (peak fraction 62), and FVa (peak fraction 70) (Fig. 5*A*) were different from full-length TFPIα (peak fraction 90) (Fig. 5*B*). Preincubation of FV-short with TFPIα (1 μM, each, Fig. 5*C* or 50 nM each, data not shown) showed that TFPIα coeluted with FV-short on the SEC column suggesting that the proteins form a complex. These data also show that anionic phospholipids are not required for FV-short binding to TFPIα. FV-short precleaved at Arg^709^ (FV-short-RQ∗) bound TFPIα (Fig. 5*D*) while precleavage at Arg^1545^ (FV-short-QR∗) eliminated binding (Fig. 5*E*); FVa did not detectibly bind TFPIα using this method (Fig. 5*F*). In contrast to FV-short, TFPIα eluted as a separate peak when mixed with PD-FV or FV-1033 suggesting the lack of an interaction between the proteins (Fig. 5, *G* and *H*). These data suggest that, at physiologic concentrations (FV, 20 nM and TFPIα, ∼0.25 nM), FV and TFPIα are unlikely to form a complex in plasma.

**Figure 6:**
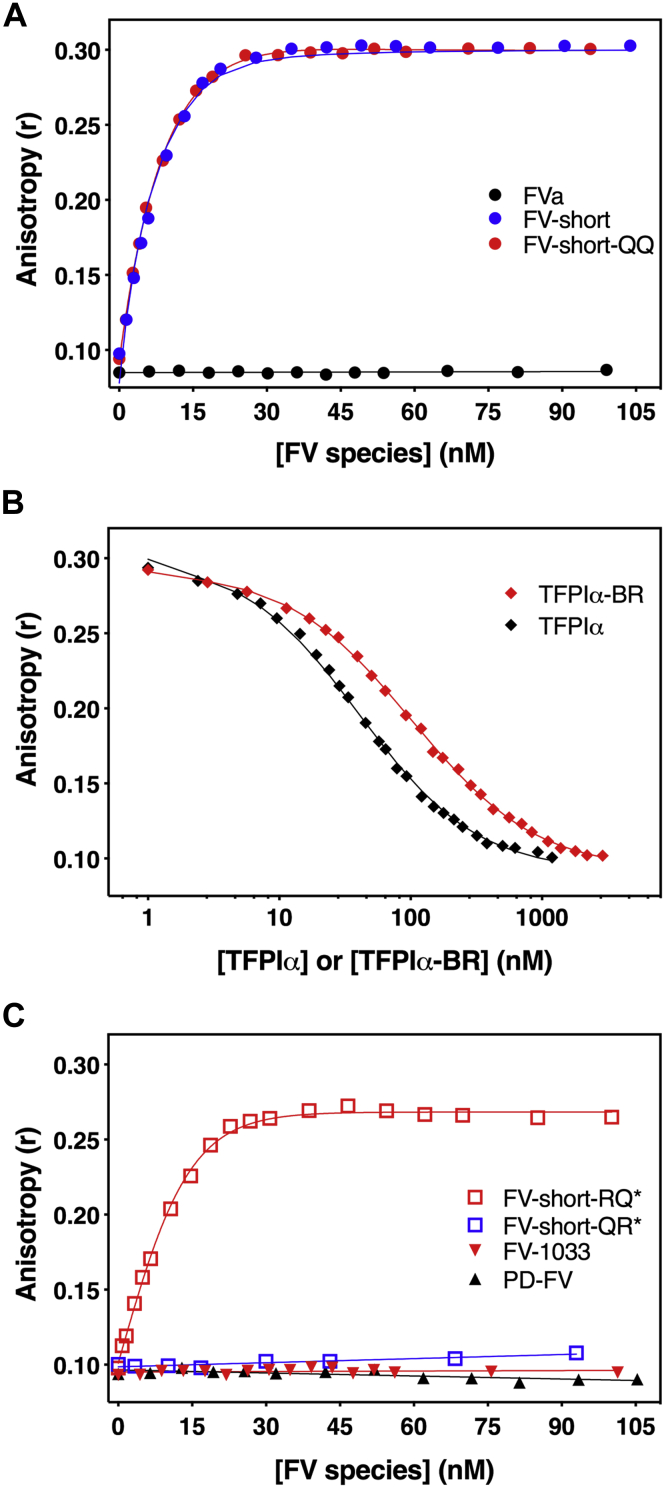
**TFPIα-BR binds to FV-short with high affinity.***A* and *C*, reaction mixtures containing 20 nM OG_488_-TFPIα-BR and 50 μM PCPS in assay buffer were titrated with increasing concentration of FV species. The (∗) refers to FV-short mutants precleaved prior to introduction into the assay. The lines are drawn following analysis to independent, noninteracting sites. *B*, unlabeled TFPIα-BR or TFPIα was titrated into reaction mixtures containing 25 nM OG_488_-TFPIα-BR, 20 nM FV-short-QQ, and 50 μM PCPS. Equilibrium binding constant was determined assuming a stoichiometry of 1 mol of FV-short-QQ/mol of BR peptide. Data are representative of 2 to 4 similar experiments.

We also measured binding by monitoring changes in anisotropy using a fluorescently labeled TFPIα-BR fragment (OG_488_-TFPIα-BR). When FV-short was titrated into reactions containing a fixed concentration of OG_488_-TFPIα-BR and membranes, it produced a saturable binding curve with a calculated equilibrium binding constant of K_d_ = 0.71 ± 0.11 nM and n = 1.25 ± 0.13 (Fig. 6*A*). Similar results were obtained with FV-short-QQ, while no binding was detected with FVa (Fig. 6*A*). Competition experiments using unlabeled TFPIα-BR or full-length TFPIα showed that these proteins displaced OG_488_-TFPIα-BR from FV-short (Fig. 6*B*). The calculated affinities for binding between FV-short and unlabeled TFPIα-BR (K_d_ = 2.25 ± 0.20 nM) and TFPIα (K_d_ of 0.73 ± 0.16 nM) were similar to OG_488_-TFPIα-BR. These data show that Kunitz domains (K1, K2, and K3) of TFPIα do not significantly impact binding to FV-short.

Consistent with functional measurements and SEC data, OG_488_-TFPIα-BR also bound to FV-short cleaved at Arg^709^, albeit with a slightly reduced affinity (K_d_ = 2.16 ± 0.25 nM) (Fig. 6*C*). Cleavage of FV-short at Arg1545 eliminated binding to OG_488_-TFPIα-BR, again highlighting the critical importance of AR2 being attached to the LC (Fig. 6*C*). Moreover, we were unable to detect binding of PD-FV or FV-1033 to OG_488_-TFPIα-BR. This, along with the SEC data, provides two independent methods showing that TFPIα does not directly bind full-length, uncleaved FV. If binding occurs, it is weak (μM range) and unlikely to be of physiologic importance.

### Cleavage of FV at Arg^709^ and Arg^1018^ enables binding to TFPIα-BR

Our data suggest that when FV-short is introduced into assay systems in which thrombin or FXa is produced, its sensitivity to TFPIα changes over time due to cleavage within the B-domain. To test this directly, we mixed equal molar amounts of OG_488_-TFPIα-BR and FV-short and added catalytic amounts of thrombin. Changes in fluorescence anisotropy were monitored over time, and aliquots were taken to assess FV-short cleavage.

**Figure 7:**
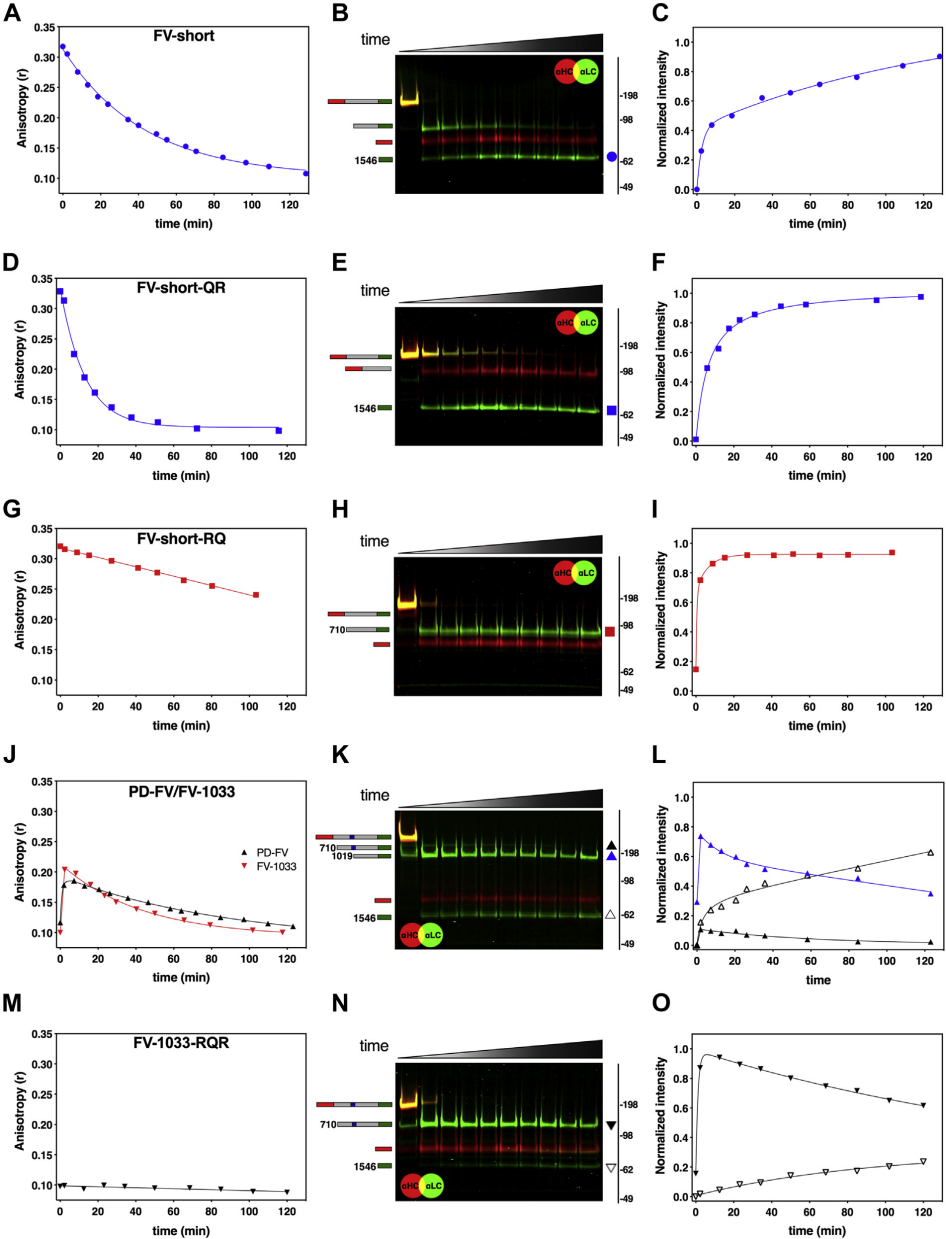
**Cleavage of FV facilitates binding to TFPIα-BR.** FV species (40 nM each; *A*, *D*, *G*, *J* and *M*) were added to a cuvette containing 50 μM PCPS and 40 nM OG_488_-TFPIα-BR in assay buffer. In *A*, *D*, *G*, *J* and *M*, the steady-state fluorescence anisotropy change was measured over time following the addition of thrombin. At the same time intervals, aliquots were collected over time and subjected to SDS-PAGE/western blotting on 3 to 8% gradient gels under reducing conditions, and the FV fragments were visualized using fluorescently labeled antibodies directed against the FV-LC (*green*) and the FV-HC (*red*) (*B*, *E*, *H*, *K* and *N*). FV species corresponding to each band are schematically shown on the left of the blots: HC (*red bar*), B-domain (*gray bar*), LC (*green bar*), and BR (*blue bar*). Symbols to the right of the blot (*B*, *E*, *H*, *K*, *N*) representing FV fragments were plotted as normalized intensity on the graphs to the right and match in color and shape (*C*, *F*, *I*, *L*, *O*). The data are representative of 2 to 3 similar experiments.

Following addition of thrombin, the high anisotropy signal resulting from FV-short binding to OG_488_-TFPIα-BR decreased over time (Fig. 7*A*). Detection of FV-short HC and LC fragments by western blotting showed that the decrease in anisotropy correlates with the appearance of the LC due to cleavage at Arg^1545^ (Fig. 7, *B* and *C*). Similar data were obtained with FV-short-QR, which is only cleaved at Arg^1545^ (Fig. 7, *D*–*F*). In contrast, thrombin only modestly reduced the anisotropy signal of FV-short-RQ despite rapid cleavage at Arg^709^ (Fig. 7, *G*–*I*). This is likely due to the weakened affinity for TFPIα-BR seen when FV-short is cleaved at Arg^709^.

Since FV does not bind TFPIα, mixing of PD-FV or FV-1033 with OG_488_-TFPIα-BR resulted in a low background anisotropy signal (Fig. 7*J*). However, upon addition of thrombin, the signal increased suggesting cleaved forms of FV bind to OG_488_-TFPIα-BR (Fig. 7*J*). Over the time course, the signal gradually decreased to baseline levels, suggesting further proteolytic processing by thrombin-eliminated binding. Western blotting of the reaction mixture showed cleavage of FV by thrombin (Fig. 7*K* and data not shown for FV-1033). The increase in anisotropy correlated with the appearance of the 1019 to 2196 fragment, which results from FV cleavage at Arg^709^ and Arg^1018^ by thrombin (Fig. 7*L*). The slow decrease in anisotropy correlated reasonably well with cleavage at Arg^1545^. However, while the anisotropy signal returned almost to baseline, there was still a significant fraction of FV not cleaved at Arg^1545^. We speculate that this discordance relates to competition between OG_488_-TFPIα-BR (40 nM) and the liberated FV B-domain fragment (Arg^710^–Arg^1018^), which harbors the FV-BR and would be increasing in concentration during the measurement (∼40 nM).

Since FV was rapidly cleaved at Arg^709^ and Arg^1018^, we could not discriminate the role of these individual cleavage sites on TFPIα-BR binding. To address this, we used an FV-1033 mutant that cannot be cleaved at Arg^1018^. Despite the addition of thrombin, FV-1033-RQR did not detectably bind OG_488_-TFPIα-BR as the anisotropy signal remained at baseline levels over time (Fig. 7*M*). Western blotting revealed that FV-1033-RQR was cleaved by thrombin at Arg^709^ and Arg^1545^, but this had no correlation to OG_488_-TFPIα-BR binding (Fig. 7, *N* and *O*). These data importantly show that single cleavage of FV at Arg^709^ has no impact on binding of FV to TFPIα-BR. We speculate that this is related to the fact that the BR within the FV B-domain remains covalently attached and engaged intramolecularly with AR2. Our data show that only when FV is processed at Arg^1018^ can TFPIα-BR engage FV and interact with its newly available AR2. Overall the data show that TFPIα does not bind FV, but rather binds proteolytic intermediates of FV with a liberated BR.

### Intramolecular FV BR binds with high affinity to AR2

**Figure 8:**
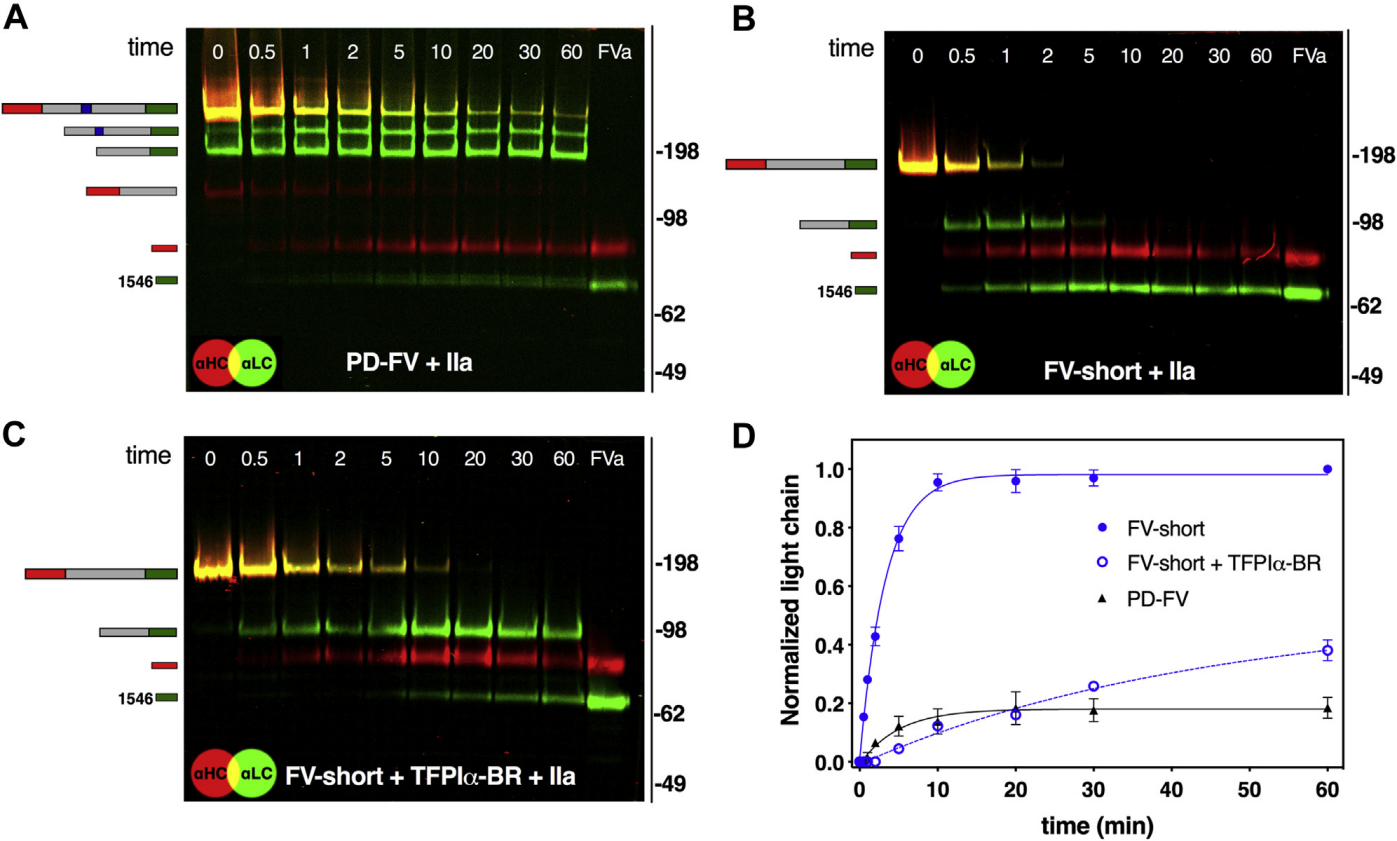
**The endogenous FV BR and TFPIα-BR delay FV cleavage by thrombin.** Twenty nanometer PD-FV (*A*) or FV-short in the absence (*B*) or in the presence (*C*) of 1 μM TFPIα-BR in assay buffer was incubated with thrombin (0.2 nM). Samples collected over time (indicated on blots in minutes) were subject to SDS-PAGE/western blotting on 3 to 8% gradient gels under reducing conditions and visualized using a mixture of fluorescently labeled antibodies against the FV-LC (*green*) and FV-HC (*red*). Species corresponding to each band are drawn on the left side: HC (*red bar*), B-domain (*gray bar*), BR (*blue bar*). LC (*green bar*). *D*, normalized intensity of the formation of the LC for PD-FV or FV-short in the presence or absence of TFPIα-BR was plotted as function of time. Data show the average of three independent experiments with standard deviation.

In addition to blocking FV-short function, we noticed that TFPIα-BR slows the rate of FV-short cleavage by thrombin, particularly at Arg^1545^; an observation reported by others (28). This finding provided an opportunity to gauge the relative affinity of the intramolecular interaction between FV BR and AR2 compared with the intermolecular interaction between TFPIα-BR and AR2 on FV-short. As shown in Figure 8, *A* and *B*, thrombin cleaves FV-short more rapidly than FV. This is particularly evident when comparing the rate of LC formation between FV and FV-short (Fig. 8*D*). FV-short and FV HC cleavage appears comparable. For FV, the data are consistent with prior work showing that cleavage at Arg^1545^ is rate limiting (3, 38). However, when a 50-fold molar excess of TFPIα-BR was added to the reaction, the rate of LC formation following FV-short cleavage by thrombin was similar to FV (Fig. 8, *C* and *D*). Comparable results were obtained when using membrane-bound FXa or RVV-V as the enzyme (data not shown). Further, the same delay in FV-short cleavage by thrombin was seen whether TFPIα-BR or TFPIα was used (Fig. S2). Together, these data suggest that for FV, the endogenous BR binds to AR2 at least 50-fold tighter compared with the binding of TFPIα-BR to FV-short. While this is likely an underestimation, the findings are in line with differences in affinity between other intramolecular *versus* intermolecular interactions (39).

## Discussion

In this study, we show which forms of FV directly bind and are regulated by TFPIα *via* its BR. These include FV-short and partially cleaved FV (FV_AR2_), but do not include full-length FV or FVa. These findings highlight the critical role played by the B-domain in regulating TFPIα binding. TFPIα *via* its C terminus directly impacts thrombin generation by targeting FV-short and other FV_AR2_, which could be derived from platelets or from PD-FV cleaved at both Arg^709^ and Arg^1018^ once thrombin or FXa is produced. All forms of FV that have an accessible AR2 bind TFPIα-BR, which effectively acts as surrogate for the endogenous FV BR, keeping these forms of FV in a procofactor state.

**Figure 9:**
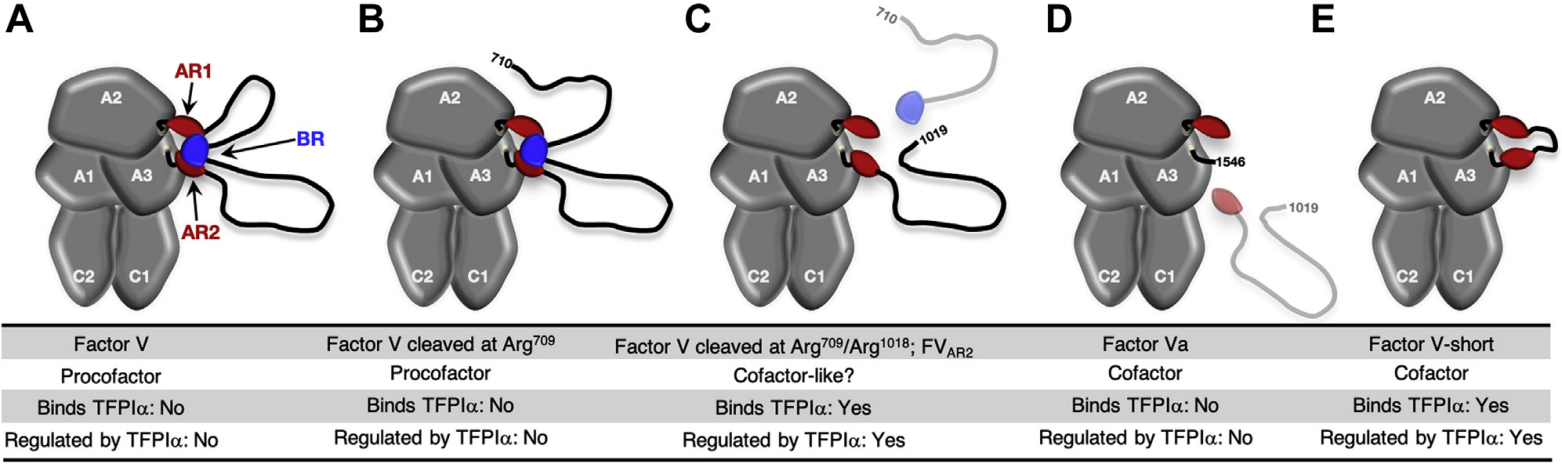
**Properties of different forms of FV and regulation by TFPIα.***A*, FV is a procofactor and is maintained in an inactive state by the concerted action of the BR and AR2. These functional landmarks likely interact with AR1 and block the ability of FXa to bind on the HC/LC region. Due to the unavailability of AR2, TFPIα does not bind FV and is not regulated by it directly. *B*, cleavage of FV at Arg^709^ by thrombin or FXa has little to no influence on weakening the interaction of BR with AR2. *C*, further cleavage at Arg^1018^ disrupts BR binding and generates a FV species (FV_AR2_) that has cofactor-like activity, which can bind TFPIα and be regulated by it. *D*, cleavage at Arg^1545^ to generate FVa, releases AR2, and eliminates TFPIα binding. *E*, since FV-short lacks the BR yet retains AR2, it is cofactor-like FVa, but can bind and be regulated by TFPIα.

The BR and AR2 of FV represent critical functional landmarks that interact with each other and are the minimal B-domain sequences needed to enforce the FV procofactor state (Fig. 9*A*) (2, 23). With these intramolecular interactions, it is not surprising that we could not detect binding of FV to TFPIα. The TFPIα-BR simply cannot compete with covalently attached BR for binding to AR2, which has a strong apparent intramolecular affinity (Fig. 8). TFPIα binding was only detected when FV was cleaved at both Arg^709^ and Arg^1018^ (Fig. 9*C*). The dissociation of this fragment would expose AR2 to TFPIα-BR. However, it is likely that there is competition between TFPIα-BR and the liberated Arg^710^-Arg^1018^ fragment, which contains FV-BR. Depending on concentrations, the latter fragment could reengage FV_AR2_ and block TFPIα-BR binding and also suppress cofactor function. Due to this, assessing the extent to which cleaved FV (*e.g.*, FV_AR2_) is regulated or not by TFPIα-BR is complicated. We were able to show, however, that no binding could be detected if FV was cleaved only at Arg^709^ (Fig. 9*B*) although this cleavage may marginally weaken the interaction. This suggests that the BR remains associated with AR2 after cleavage at Arg^709^ and likely dissociates following cleavage at Arg^1018^. Importantly, and consistent with other work (17, 18), cleavage at Arg^1545^ removes AR2 and generates FVa that does not bind TFPIα or TFPIα-BR and is not regulated at the level of prothrombinase by these molecules (Fig. 9*D*). This model is based on both functional assays and direct binding measurements. The problem with relying only on activity measurements is that it is difficult to ascertain which form of FV (FV/FV_AR2_ or FV-short) binds TFPIα as FV changes during any assay that produces FXa or thrombin. We also found that FV-short is constitutively procoagulant in the absence of proteolysis as it functions in the prothrombinase complex like FVa (Fig. 9*E*). FV-short binds tightly to TFPIα, and this interaction blocks FV-short constitutive procoagulant activity. These observations suggest that FV-short, the only known single-chain FV_AR2_, is the main physiologic ligand of TFPIα.

Moreover, our fluorescence measurements paired with western blotting document for the first time a direct assessment of the forms of FV/FV-short that bind to TFPIα following their proteolytic transformation by thrombin (Fig. 7). The results with FV were particularly interesting as the data show that cleavage at Arg^709^ and Arg^1018^ is necessary and sufficient for TFPIα-BR binding. It is certainly possible, however, that proteins or other highly charged molecules could bind FV and disrupt/weaken the BR–AR2 interaction. This could effectively “activate” FV without proteolysis and/or make FV available to engage with TFPIα.

Our data with FV and TFPIα contrasts with prior work. However, the discovery of FV-short and other technical considerations may account for some of those prior findings. For example, immunodepletion studies showing removal of both FV and TFPIα with FV antibodies did not account for FV-short in the plasma as it was discovered years after these reports (24, 25). FV-short, estimated to be at about the same concentration of TFPIα in plasma (31), was undoubtedly immunodepleted in these studies as either a monoclonal antibody targeting the HC (24, 40) or a polyclonal antibody was used (25). SPR spectroscopy measurements with FV and immobilized TFPIα reported a K_d_ of 13 nM (24); however, this seems very unlikely based on the data presented here. If the binding affinity was in this range, we would have expected to detect binding between FV and TFPIα in chromatography (Fig. 5) or fluorescence experiments (Fig. 6). We speculate that the apparent moderate affinity interaction reported previously could reflect the fact that even the best preparations of purified PD-FV contain a certain amount of poorly defined shorter FV molecules, which could be FV-short or cleaved forms of FV (*e.g.*, FV_AR2_) or both. These forms of FV will contribute to overall binding especially using a method such as SPR spectroscopy.

TFPIα is reported to be mostly bound to FV-short in the plasma using immunoprecipitation methods with only small amounts associated with FV (33). These data suggest that TFPIα binds with a much higher affinity to FV-short compared with FV, consistent with our binding measurements. However, it was not clear based on these prior qualitative studies how much FV was associated with TFPIα and whether it interacts with its BR or another domain. As noted, the high-affinity interaction of the FV BR with AR2 makes it very unlikely that TFPIα could displace FV BR. It is possible that the experimental conditions employed or other accessory plasma proteins (*e.g.*, protein S) could have influenced previous observations. It is important to note that based on the plasma concentration of FV (20 nM), FV-short (<0.2 nM), and TFPIα (∼0.25 nM), and the dissociation constants reported here, about half of the TFPIα would be bound to FV-short while essentially none would be bound to FV. However, TFPIα is also associated with the endothelium potentially complicating these assumptions, and protein S is known to interact with both FV and TFPIα, which could also influence results (41).

FV deficiency is associated with low plasma levels of TFPIα, and the concentration of these proteins appears to correlate in the plasma (24). This is a very important finding as it potentially explains the milder than expected bleeding phenotype (24, 42). While this would seem to suggest that FV and TFPIα may interact in the plasma, again this was before knowledge of FV-short. We hypothesize that this correlation reflects the correlation between FV and FV-short levels. It is very likely that FV deficiency would also result in FV-short deficiency providing a reasonable explanation for the prior findings. While this remains to be firmly established, it highlights the need to develop an assay to assess FV-short protein levels in the plasma.

TFPIα directly regulates thrombin generation by binding cleaved forms of FV with an available AR2 and blocking their assembly into prothrombinase (18). For plasma FV, this requires cleavage at Arg^709^ and Arg^1018^ during the initiation of coagulation. Since platelet FV is stored and released from α-granules in a partially proteolyzed state (43, 44), it could also be regulated by TFPIα. Based on the plasma concentrations of FV, FV-short, and TFPIα, it is unlikely that cleaved PD-FV is regulated by TFPIα *in vivo*, although this remains to be determined. Rather, TFPIα likely regulates FV-short and platelet-FV. The latter is especially interesting due to the fact that TFPIα is also released from platelets following activation (21, 45, 46). We think the binding and regulation of FV-short by TFPIα are particularly important considering it has constitutive procoagulant activity. Cleavage of FV-short at Arg^1545^ relieves this inhibition, yet this site is protected somewhat from thrombin attack due to the TFPIα-BR interaction with AR2 (28). Based on work over the past several years, it is clear that in various purified or plasma-based assays, the inhibitory effects of TFPIα-BR on thrombin generation are observed (18, 28). What remains to be established is whether TFPIα regulation of FV_AR2_/platelet-FV/FV-short is physiologically relevant *in vivo*. This is an important question to answer as it could open up opportunities to target these interactions to enhance/suppress thrombin generation. It could also be relevant as there are antibodies in clinical development for hemophilia that block TFPI function, which may or may not influence these molecular interactions (47).

The FV/FV-short–TFPIα interaction has also been reported to influence the inhibition of FXa, most likely by promoting TFPIα binding to phospholipid (27, 31, 48). While it was originally found that FV could enhance the activity of TFPIα (19, 26), more recent work indicates that it is the combined effect of FV and protein S that is critical (27). Two studies have now shown that FV alone does not influence TFPIα-mediated FXa inhibition (27, 37). Similarly, FV-short alone did not stimulate TFPIα but did so in the presence of protein S and was a much more efficient cofactor for TFPIα compared with FV (37). These collective results are very interesting and suggest that a key function of FV-short may be to enhance the activity of TFPIα in the regulation of the initiation of coagulation by inhibiting FXa. At present, the role of cleaved FV or platelet-derived FV in stimulating this activity is not known.

In summary, we have identified the mechanism by which TFPIα *via* its C-terminal BR interacts with and regulates different forms of FV including FV-short. FV does not bind (or binds very weakly) to TFPIα and is not directly regulated by it. Rather, FV-short is the key physiologic ligand for TFPIα. This serves multiple roles including suppressing FV-short procoagulant function, complex formation in plasma, and enhancement of TFPIα toward FXa. The FV-short–TFPIα interaction promotes anticoagulation until FV-short is cleaved at Arg^1545^. However, it remains to be determined what role this interaction has in normal hemostasis. What is clear is that the unveiling of AR2 on different forms of FV in the blood (*e.g.*, FV, FV-short, FVa, platelet-derived FV, and FV_AR2_) dictates TFPIα binding and joint regulation. We speculate that strategies targeting the interaction of TFPIα with these different forms of FV may be an attractive way to either enhance or dampen the coagulation system.

## Experimental procedures

### Reagents

H-D-phenylalanyl-L-pipecolyl-L-arginyl-p-nitroanalide (S-2238) was from Diapharma Group, Inc (West Chester, OH), and its concentration was verified using ε_342_=8270 M^−1^ cm^−1^ (49). Z-glycine-glycine-arginine-AMC (I-1140) was from Bachem (Bubendorf, Switzerland). Benzamidine, aprotinin, 4-amidinophenylmethanesulfonyl fluoride hydrochloride (APMSF), isopropyl β-D-1-thiogalactopyranoside (IPTG), and bovine serum albumin (BSA) were from Sigma (St Louis, MO). Human plasma used for the isolation of coagulation proteins was a gift from the plasmapheresis unit of the Hospital of the University of Pennsylvania. Dansylarginine-N-(3-ethyl- 1,5-pentanediyl)amide (DAPA), D-Phenylalanyl-prolyl-arginyl Chloromethyl Ketone (P-PACK), Russell’ viper venom FV activator (RVV-V), and antibodies directed against the HC (AHV-5146) and the LC (AHV-5112) of FV were from Haematologic Technologies (Essex Junction, VT). The N-hydroxysuccinimidyl esters IRDye 680LT and N-hydroxysuccinimidyl esters IRDye 800CW were from LI-COR Biosciences (Lincoln, NE) and Oregon Green_488_ maleimide (OG_488_) was from Life Technology (Carlsbad, CA). All tissue culture reagents were from Invitrogen (Carlsbad, CA), except insulin-transferrin-sodium selenite (ITS) (Roche - Indianapolis, IN). Chemically competent BL21(DE3) cells were from EMD Millipore, (Billerica, MA). TFPIα was expressed in *E. coli* (rh-TFPI, Tifacogin, Novartis). Verification of an intact C-terminal basic region was confirmed by western blot analysis (data not shown). The mouse antihuman monoclonal antibodies directed against the K2 domain of TFPIα (clone M105272) were from Fitzgerald Industries International (Acton, MA). Pooled normal plasma (PNP) and FV-deficient plasma (DP) were from George King Bio-medical Inc (Overland Park, KS) and the PT reagent (TriniClot PT Excel) was from Tcoag (Wicklow, Ireland). Dade Innovin was from Siemens Healthcare (Marburg, Germany), and the TF concentration was assumed to be 6 nM once reconstituted in 10 ml of water as recommended. Small unilamellar phospholipid vesicles (PCPS) composed of 75% (w/w) hen egg L-α-phosphatidylcholine and 25% (w/w) porcine brain L-α-phosphatidylserine (Avanti Polar Lipids, Alabaster, AL) were prepared and characterized as previously described (50). Unless specified, all functional assays were performed at pH 7.4 in 20 mM HEPES, 0.15 M NaCl (HBS), 5 mM CaCl_2_, and 0.1% (w/v) polyethylene glycol 8000 (assay buffer).

### Proteins and peptides

Plasma-derived FV was isolated from 4 L of plasma by immunoaffinity chromatography followed by ion-exchange chromatography as previously described (38, 51, 52). FX and prothrombin were purified from human plasma as described (52, 53, 54), and FXa and thrombin were prepared following preparative activation of the respective zymogens and purified (38, 54, 55). Recombinant FV-short and thrombin-resistant mutants in which Arg (Arg^709^ and/or Arg^1545^) was replaced with Gln (FV-short RQ, FV-short-QR, FV-short-QQ) FV-1033, FV-1033-RQR were expressed in BHK cells and purified as described (14, 15). FVa was prepared following treatment of FV-short with thrombin and purified as previously described (14, 56). FV-BR, TFPIα-BR, and TFPIα-BR(Cys) (containing an N-terminal cysteine) peptides were expressed in *E. coli* and purified as previously described (17, 18). OG_488_-TFPIα-BR was prepared by coupling OG_488_ to TFPIα-BR(Cys) as previously described (17, 18), and its concentration was determined using *ε*_491_ = 81,000 M^−1^ cm^−1^. Molecular weights (M_r_) and extinction coefficients ($E_{280\;}^{0.1\%}$) of the various proteins as well as FV-BR and TFPIα-BR have been previously reported (16, 17, 18). FV-short and FV-short-QQ protein concentrations were determined using values previously determined for FV-810 (M_r_ = 216 kDa and an $E_{280\;}^{0.1\%}=\;1.54$) (14). For experiments requiring precleavage, FV-short-RQ (300 nM) in assay buffer was incubated with 2 nM thrombin (defined as FV-short-RQ∗), and FV-short-QR (300 nM) in assay buffer was incubated with 2 nM RVV-V (defined as FV-short-QR∗) for 30 min at 37 °C. The reaction with thrombin was quenched with 4 nM hirudin. Purity was assessed by SDS-PAGE under reducing conditions (50 mM dithiothreitol) using 4 to 12% precast gradient gel (Invitrogen) using the MOPS (proteins) or MES (peptides) buffer system followed by staining with Coomassie Brilliant Blue R-250. Antibodies directed against the HC (AHV-5146) and the LC (AHV-5112) of FV were labeled with N-hydroxysuccinimidyl esters IRDye 680LT and with N-hydroxysuccinimidyl esters IRDye 800CW respectively as previously described (38). Briefly, 10 μM antibody and 50 μM probe in 20 mM HEPES, 0.15 M NaCl, pH 7.8 were incubated for 3 h at 25 °C, and the reaction was quenched by the addition of 10 mM Tris, pH 8.0. Labeled antibodies were purified from excess probe by centrifugal gel filtration using P6-DG (Bio-Rad) equilibrated in 20 mM HEPES, 0.15 M NaCl, pH 7.5.

### Clotting and thrombin generation assay

For clotting assays, FV species (100 nM) in assay buffer were diluted to 0.4 nM in HBS with 0.1% BSA. The specific clotting activity was measured at 37 °C in FV-DP supplemented with 0.2 nM FV species in the presence or absence of 5 μM BR fragments and initiated with TriniClot PT Excel as previously described (57). TGA was measured with a fluorometric assay in the plasma using a modified protocol (58, 59). Either FV-DP reconstituted with 20 nM FV or 1.5 nM FVa/FV-short mutants or PNP was preincubated for 10 min with different concentrations of TFPIα-BR (0–6 μM). Thrombin generation was triggered by Innovin (TF concentration of 0.4 or 5 PM) or FXa (200 PM) and PCPS (4 μM final) and initiated immediately by adding the fluorogenic substrate I-1140 in 15 mM CaCl_2_. Fluorescence was measured at 37 °C using the excitation and emission wavelengths of 360 and 460 nm respectively for 90 min using a Molecular Devices Spectromax M2 plate reader.

### Prothrombin activation assay

Steady-state initial velocities of prothrombin activation were determined discontinuously in assay buffer at 25 °C as described (14, 17, 54, 60). The catalytic constants K_M_ and V_max_ were determined using the following reaction conditions: PCPS (50 μM), prothrombin (0–1.4 μM), and DAPA (3 μM) were incubated with FVa or FV-short mutants (20 nM) in assay buffer, and the reaction was initiated with FXa (0.1 nM). At various time points over 3 min, aliquots of the reaction mixture were quenched in buffer containing 50 mM EDTA, and thrombin generation was determined using the chromogenic substrate S-2238. The effect of BR peptides and TFPIα on the prothrombinase activity was measured similarly using the following reaction conditions: PCPS (50 μM), prothrombin (1.4 μM), DAPA (3 μM), and FVa or FV-short species (0.1 nM) were incubated with BR peptides (0–7 μM) or TFPIα (0–500 nM) supplemented with a twofold excess of anti-K2 antibody (0–1 μM). The reaction was initiated with 2 nM FXa.

### Gel filtration chromatography

A 10 × 1000 mm HR Sephacryl S-200 column (Kimble, USA) equilibrated with HBS and 5 mM CaCl_2_ (HBS/Ca^2+^, pH 7.4) was loaded with 500 μl of 1 μM TFPIα or FV species alone or with 1 μM TFPIα/1 μM FV species preincubated at 25 °C for 10 min. Fractions (550 μl; 100 μl/min flow rate; 4^o^C) were collected and monitored by UV absorbance at 280 nm for FV-species and for TPFIα using Asserachrom Total TFPI ELISA kit (Stago; Parsippany, NJ) according to the manufacturers’ protocol. TFPIα used for the experiments and TFPIα from the ELISA kit produced similar results when used as standards (0–200 ng/ml). In control experiments, FV-short and TFPIα did not interact when run using HBS and 20 mM EDTA (data not shown).

### Fluorescence anisotropy measurements

Steady-state fluorescence anisotropy was measured in a QuantaMaster spectrophotometer (Photon Technology International; Birmingham, NJ) using excitation and emission wavelengths of 480 and 520 nm, respectively, with long-pass filters (KV500, CVI Melles Griot) in the emission beam. All fluorescence anisotropy measurements were carried out in a 1 cm^2^ quartz cuvette at 25 °C in assay buffer containing 50 μM PCPS as previously described (17, 18, 61). For direct binding measurement, increasing concentrations of FV species (0–100 nM) were added to the reaction mixtures (2.5 ml) containing 20 or 40 nM OG_488_-TFPIα-BR. For competition experiments, TFPIα-BR (0–2 μM) or TFPIα (0–1 μM) was titrated into the cuvette containing OG_488_-TFPIα-BR and FV-short-QQ. For experiments in which FV species were cleaved, catalytic amount of thrombin (or RVV-V for FV-short-QR) was added to a cuvette in which 40 nM OG_488_-TFPIα-BR was preincubated with 40 nM FV species and changes in fluorescence anisotropy were followed over time. Timed aliquots taken from the activation mixture were used for western blotting analysis.

### Proteolysis of PD-FV and FV-short

PD-FV or FV-short (20 nM) alone or supplemented with 1 μM TFPIα-BR was prewarmed at 37 °C for 5 min. Thrombin (0.2 nM) was added, and aliquots collected over 60 min were assessed by western blotting analysis. FV-short cleavage by thrombin after 15 min was also assayed in the presence of increasing concentrations (0–5 μM) of TFPIα-BR or TFPIα.

### Western blot and densiometric analysis

Protein samples were reduced and denatured at 80 °C for 7 min and electrophoresed on 3 to 8% polyacrylamide gels in Tris-Acetate buffer. Gels were directly blotted following the LI-COR protocol with some changes. Briefly, gels were fixed in 50% isopropanol, 5% acetic acid solution and then blocked for 30 min in PBS 5% BSA 0.1% Tween-20. Protein bands were visualized using a mixture of antibodies against the HC (AHV-5146 labeled with IRDye 680LT) and the LC (AHV-5112 labeled with IRDye 800CW). Blots were scanned (Odyssey, LI-COR) using two channels (700 nm for HC antibody and 800 nm for LC antibody). Signal intensity of each band was assessed using the Image Studio Lite software (LI-COR).

### Data analysis

Steady-state kinetic constants were determined by nonlinear least squares analysis according to the Michaelis–Menten equation. Inhibition constant (K_i_) was determined under steady-state condition using the equation for competitive inhibition. Equilibrium dissociation constants (K_d_), stoichiometries (n), and the maximum increase in anisotropy at saturating FV species concentrations were obtained for the interaction between TFPIα-BR and FV species from the dependence of the anisotropy with increasing concentrations of FV species and were corrected for the overall change in fluorescence intensity (62, 63). Competition experiments, in which unlabeled TFPIα-BR or TFPIα was titrated into preformed complexes of OG488-TFPIα-BR and FV-short-QQ, were analyzed to determine K_d_ as described (17, 61). Normalization and validation of the western blotting signal were done using considerations described previously (64, 65).

## Data availability

Data for all figures are contained within the article. Additional data backing the kinetic analyses in the tables are available from the corresponding author upon request.

## Conflict of interest

R Camire is a consultant for and receives research support from Pfizer and Bayer.

## Footnotes

†† FV activity in normal plasma is 1 Unit/ml = 50 Units/nmol.
