# Supplementary material for: Regulation of factor V and factor V-short by TFPIα: Relationship between B-domain proteolysis and binding
Source: J Biol Chem. 2021 Jan 7;296:100234. doi: 10.1074/jbc.RA120.016341 (PMC7948760; doi:10.1074/jbc.RA120.016341)
Supplement: Supplemental Figures S1 and S2 [file mmc1.pdf]

Supporting information:

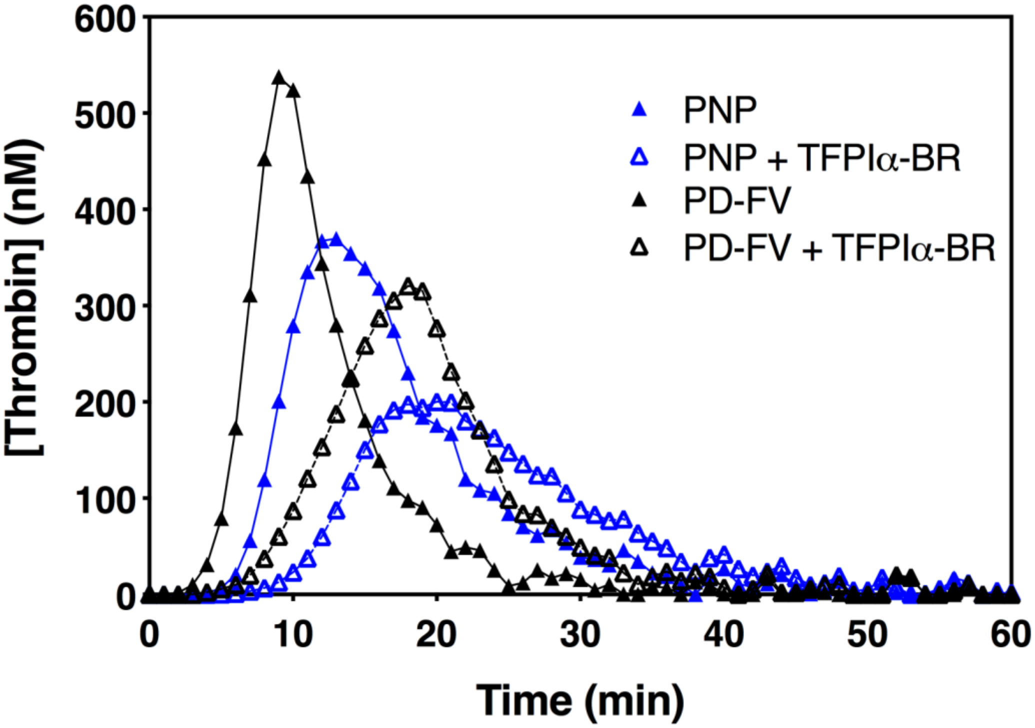

Supporting information 1. TFPIα-BR blocks thrombin generation in the presence of FV. Thrombin generation for PNP or FV-DP supplemented with 20 nM PD-FV in the presence or absence of TFPIα-BR (6 μM) using 0.4 pM TF as the initiator. Data are representative of 3 independent experiments.

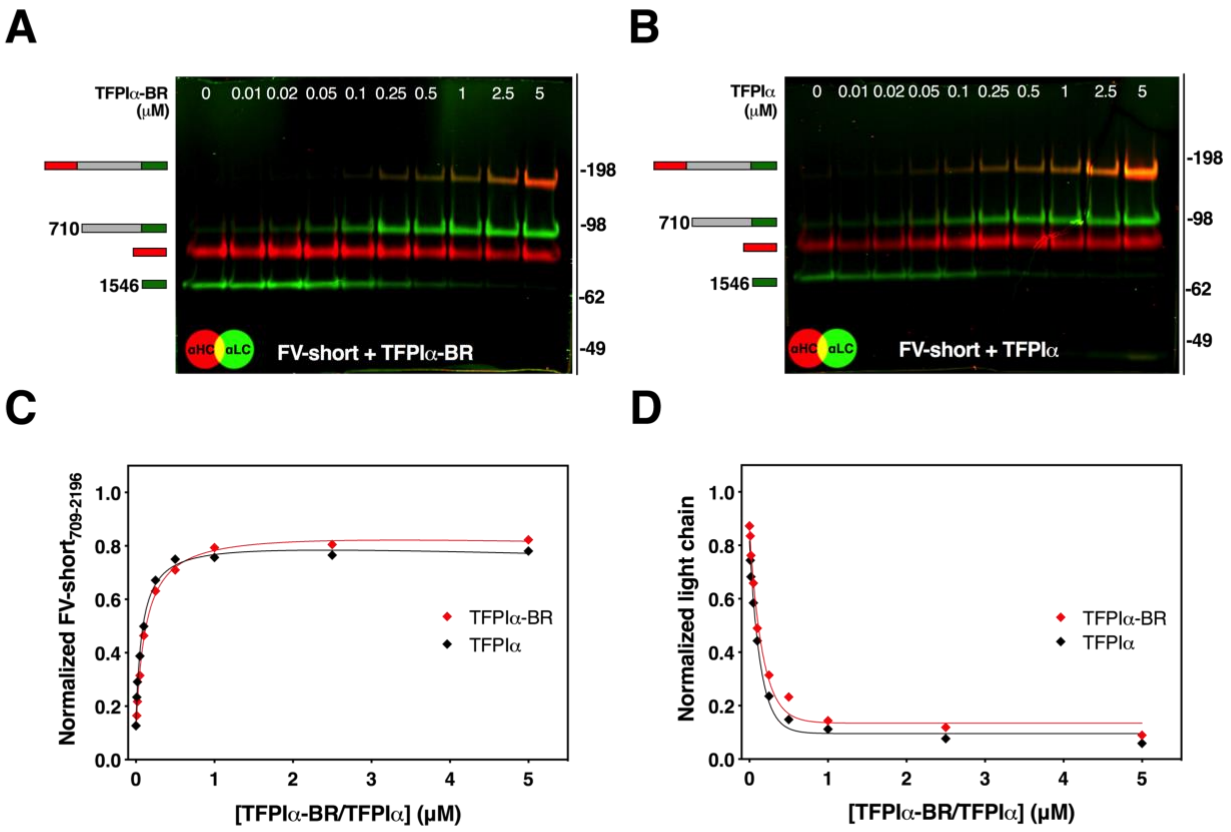

Supporting information 2. TFPIα-BR or TFPIα delays FV-short activation by thrombin. FV-short (20 nM) was incubated for 15 min with thrombin (0.2 nM) in the presence of increasing concentrations (0-5 μM; indicated on the blot) of TFPIα-BR (*A*) or TFPIα (*B*). Aliquots collected after 15 minutes were subject to SDS-PAGE/western blot on 3-8% gels under reducing conditions and visualized using a mixture of labeled monoclonal antibodies against the FV-LC (green) and FV-HC (red). FV-short fragments corresponding to each band are drawn on the left side: HC (red), B-domain (grey), LC (green). Normalized intensity of the FV-short 710-2196 fragment corresponding to cleavage at Arg^{709} (shown in *C*) or FV-short fragment 1546-2196 corresponding to cleavage at Arg^{1545} (shown in *D*) were plotted as function of TFPIα-BR or TFPIα concentration. Data are representative of 3 similar experiments.
